# Description of *Streptococcus thalassemiae* sp. nov., a Bacterium Isolated from Human Blood

**DOI:** 10.1155/2023/3802590

**Published:** 2023-08-01

**Authors:** Fatou Samba Diouf, Mamadou Beye, Mapenda Gaye, Babacar Mbaye, Stephane Alibar, Mariema Sarr, Gregory Dubourg, Jean-Christophe Lagier, Cheikh Sokhna, Florence Fenollar, Pierre-Edouard Fournier, Cheikh Ibrahima Lo

**Affiliations:** ^1^Aix Marseille University, IRD, AP-HM, MEPHI, Marseille, France; ^2^IHU-Mediterranean Infection, Marseille, France; ^3^Aix Marseille University, IRD, AP-HM, SSA, VITROME, Marseille, France; ^4^Campus Commun UCAD-IRD of Hann, Dakar, Senegal

## Abstract

Blood is a precious biological liquid that is normally sterile. Therefore, bacteria in the bloodstream are shown a priori anomaly. A blood culture is systematically performed to diagnose the cause of the bacteremia. Indeed, a patient received in our service had a thalassemia major and underwent a genoidentical transplant. Then, a blood test was performed to diagnose a four-day fever. In this context, we have isolated strain Marseille-Q2617 from the blood sample. It revealed a new bacterial strain that belongs to the genus *Streptococcus.* It is a Gram-positive coccus, nonmotile, and nonspore forming. The major fatty acid found is hexadecanoic acid, with 49.5%. A taxonomic method was used to characterize the strain by studying their phenotypic, phylogenetic, and genomic characteristics. In addition, sequence analysis of the 16S rRNA gene shows that the strain Marseille-Q2617 has 99.94% sequence similarity to *Streptococcus mitis*. Average nucleotide identity (ANI) analysis for strain Marseille-Q2617^T^ showed the highest similarity of 92.9% with *S. mitis*. The DNA-DNA hybridization value obtained (50.2%) between strain Marseille-Q2607 and *S. mitis*, its closest related species, was below the recommended threshold (<70%). Strain Marseille-Q2617^T^ has a genome size of 2.02 Mbp with 40.5 mol% of G + C content. Based on these results, we propose a new species of the genus *Streptococcus*, for which the name *Streptococcus thalassemiae* sp. nov., Marseille-Q2617^T^ (=CSUR Q2617 = CECT 30109) was proposed.

## 1. Introduction

Streptococci are most frequently found in the commensal flora of the skin and upper respiratory tract [1].However, many streptococci are among the most invasive bacteria groups and appear as opportunistic pathogens in certain infections [1]. Some of these highly pathogenic species such as *Streptococcus agalactiae*, *S. gallolyticus*, and *S. pneumoniae* are involved in meningitis, endocarditis, and pneumonia pathologies [2].

*Streptococcus* is an important bacterial genus that currently includes 110 child taxa with a validly published and correct name (https://lpsn.dsmz.de/genus/streptococcus). Formerly classified into three main groups according to their hemolysis pattern (alpha-hemolytic, beta-hemolytic, and nonhemolytic or gamma-hemolytic), streptococci are presently subdivided into six groups of species based on a systematic study of the 16S rRNA gene sequence: pyogenic, anginosus, mitis, salivarius, bovis, and mutans [3, 4].

Nowadays, the general use of matrix-assisted desorption ionization time-of-flight mass spectrometry (MALDI-TOF MS) in the clinical and research environment, coupled with the rapid development of next-generation sequencing technology, gives us a new and more comprehensive insight into the taxonomy. Therefore, we have used a taxonomic approach as described [5] to provide a complete description of this new bacterium species. Thus, based on the morphological, phenotypic, biochemical, phylogenetic, and genomic features, *Streptococcus thalassemiae* sp. nov., strain Marseille-Q2617 was proposed as a new member of the genus *Streptococcus*.

## 2. Materials and Methods

### 2.1. Organism Isolation and Collection

Within the context of an investigation on the etiology of infectious diseases, samples of blood cultures were taken from patients hospitalized at the IHU mediterranean infection. The strain Marseille-Q2617 was the only one isolated bacterial strain from a patient who had major thalassemia. He had a fever (38.5°C) on day 4 of a genoidentical transplant when blood was drawn.

### 2.2. Identification of the Strain by MALDI-TOF MS and 16S rRNA Gene Sequencing

Colonies were first identified using MALDI-TOF MS on a LT Microflex spectrometer (Bruker Daltonics, Bremen, Germany) as described [6]. The obtained spectra were imported into the MALDI Biotyper 3.0 software (Bruker Daltonics) and matched against the references in the database (Bruker database incremented with in lab references (https://www.mediterranee-infection.com/acces-ressources/base-de-donnees/urms-data-base/, accessed February 2021)). The resulting score enabled (or did not enable) the identification of the tested species: colonies were labeled as correctly identified at the species level with a score ≥ 2, at the genus level with a score ≥ 1.7. Unidentified species (score < 1.7) using MALDI-TOF MS were identified using Sanger sequencing of the 16S rRNA gene. The 16S rRNA gene was amplified using the universal bacterial primers pairs fD1 and rP2 [7] (Eurogentec, Angers, France), and the resulting amplicons were sequenced using the Big Dye® Terminator v1.1 Cycle Sequencing Kit and 3500xL Genetic Analyzer capillary sequencer (ThermoFisher, Saint-Aubin, France), as described [8]. The sequences were aligned using MUSCLE with default parameters, and the phylogenetic inferences were obtained using the maximum likelihood method and the MEGA X software [9]. Bootstrap values obtained by repeating the analysis 1,000 times to generate a majority consensus tree are indicated at the nodes*. Bartonella quintana* was used as outgroup.

### 2.3. Physiology and Chemotaxonomy Characteristics

#### 2.3.1. Growth Conditions

To determine optimal conditions for strain Marseille-Q2617, several tests were performed: pH, temperatures, salinity, and atmospheres. They were cultivated on Columbia agar with 5% sheep blood (bioMerieux, Marcy l'Etoile, France) and incubated at different temperatures (28°C, 37°C, 42°C, and 56°C) and in varied atmospheres (aerobic, anaerobic, and microaerophilic) as previously described [10]. Seven pHs were tested: 5.0; 5.5; 6.0; 6.5; 7.0; 7.5, and 8. Moreover, the strain Marseille-P2915T was tested for its salinity tolerance on Columbia agar using a range of salt concentrations: 10%, 15%, 20%, and 25%.

#### 2.3.2. Biochemical, Sporulation, and Motility Tests

Biochemical substrates are tested using API ZYM (enzymatic activities), API 50 CH (carbohydrate fermentation), and API 20Strep (specific criteria) galleries. We also carried out the ability to form spores using a thermal shock. The motility assay was performed by directly examining a fresh colony using a DM 1000 optical microscope (Leica, Nanterre, France) at a ×400 magnification. Detection of catalase (bioMerieux) and oxidase activities (Becton Dickinson, Franklin Lakes, NJ, USA) was also tested.

#### 2.3.3. Antibiotic Susceptibility

Susceptibility to antibiotics was tested using Columbia agar with 5% sheep blood (bioMerieux, Marcy l'Etoile, France) and according to EUCAST 2015 recommendations (https://www.eucast.org) as described by Elsawi et al. [11]. The following antibiotics were tested: penicillin G, amoxicillin, daptomycin, ciprofloxacin, ceftazidime, ceftriaxone, clindamycin, doxycycline, fosfomycin, imipenem, linezolid, oxacillin, rifampicin, teicoplanin, tobramycin, trimethoprim‐sulfamethoxazole, and vancomycin using ETEST® (bioMerieux, Marcy l'Etoile, France).

#### 2.3.4. Electron Microscopy

The morphology of the strain was visualized with the Hitachi SU5000 scanning electron microscope (Hitachi Group, Krefeld, Germany). A colony was retrieved from agar and suspended in a 2.5% glutaraldehyde fixative solution. A drop of the suspension was then directly deposited on a poly-L-lysine coated slide for five minutes and treated with 1% phosphotungstic acid aqueous solution (pH 2.0) for two minutes to increase scanning electron micrograph (SEM) image contrasting. The slide was washed in water, air-dried, and examined using a tabletop SU5000 microscope (Hitachi High-Tech, HHT, Japan). The scale bar and acquisition settings are shown on the micrograph.

#### 2.3.5. Cellular Fatty Acid Composition

Cellular fatty acid methyl ester (FAME) analysis was performed using gas chromatography/mass spectrometry (GC/MS). Several culture plates were scraped to obtain approximately 50 mg of bacterial biomass per tube. FAMEs were prepared according to Sasser's protocol [12], and the GC/MS analyses were then done as previously described [13]. The sample was prepared with approximately 15 mg of bacterial biomass per tube harvested from several culture plates. Briefly, fatty acid methyl esters were separated using an Elite 5-MS column and monitored by mass spectrometry (Clarus 500-SQ 8 S, Perkin Elmer, Courtaboeuf, France). Spectral database search was performed using MS Search 2.0 operated with the Standard Reference Database 1A (NIST, Gaithersburg, USA) and the FAMEs mass spectral database (Wiley, Chichester, UK).

### 2.4. Genome Assembly and Analysis

The total DNA genome of the strain Marseille-Q2617 was extracted and sequenced as previously described [14]. SPAdes version 3.10.1 software [15] was used for assembling genomic reads. Mapping of reads recovered from MiSeq and MinION technologies for strain Marseille-Q2617 was carried out using CLC genomics7 (https://www.qiagenbioinformatics.com/products/). Genome strain is annotated with Prokka version 1.13.3, available in the Galaxy Australia online server (https://usegalaxy-au.github.io/). GCview software [16] was used for genome visualization. The digital DNA-DNA hybridization (dDDH) among compared genomes was calculated by the online tool genome-to-genome distance calculator, version 3.0 [17]. Furthermore, the average nucleotide identity was estimated using the Orthologous Average Nucleotide Identity Tool (OAT) [18].

## 3. Results and Discussion

### 3.1. Phylogenetic Analysis

**Figure 1:**
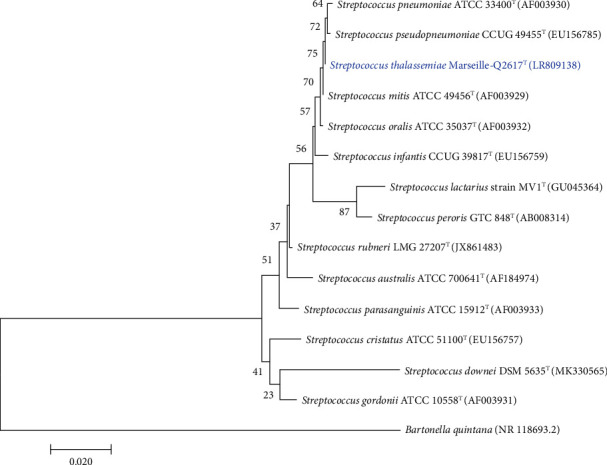
Phylogenetic tree highlighting the position of *Streptococcus thalassemiae* sp. nov., strain Marseille-Q2617 regarding others closely related species. Genbank accession numbers of 16S rRNA gene sequences are indicated in parenthesis. Sequences were aligned using MUSCLE with default parameters. Phylogenetic inferences were obtained using the maximum likelihood method and the MEGA X software [9]. Bootstrap values obtained by repeating the analysis 1,000 times to generate a majority consensus tree are indicated at the nodes*. Bartonella quintana* was used as outgroup. The scale bar represents a 2% nucleotide sequence divergence.

The spectra were added to the local Microflex database to allow future identification using MALDI-TOF MS. The phylogenetic tree based on the 16S rRNA gene highlighted the position of the Marseille-Q2617 strain in relation to all related streptococcus species whose names have been validly published in the LPSN is presented in Figure 1.

**Figure 2:**
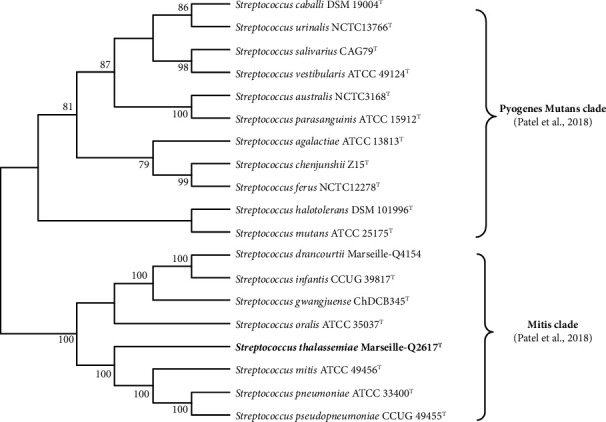
Phylogenetic tree based on five concatenated genes such as *GyrA*, *ddl*, *gdh*, *rpoB*, and *sodA* and constructed with MEGA X software. The sequences were then aligned using MUSCLE. Bootstrap values obtained by repeating the analysis 1000 times to generate a majority consensus tree are shown at nodes. Only bootstrap values above 75% are displayed. All genomic sequences of the different species studied are retrieved from the NCBI database. They are then annotated with the Prokka software. The targeted genes are extracted from genomes and concatenated with the Emboss union server (https://www.bioinformatics.nl/cgi-bin/emboss/union). This tree is in accordance with the classification of Patel and Gupta [4] and shows the separation of species into clades.

Nonidentification of the strain by MALDI-TOF mass spectrometry led us to carry out additional analysis. Currently, the 16S rRNA gene alone cannot discriminate strains of streptococci, and a polyphasic approach should be adopted [19, 20]. BLASTn was carried out on the NCBI DNA database with the sequences of the 16S rRNA genes, and strain Marseille-Q2617 shows 99.84% sequence similarity to *Streptococcus mitis* ATCC 49456 (NR_116207.1). Indeed, the sequence similarity values of the 16S rRNA gene obtained are very high compared to the threshold value recommended to delimit the species barrier [21]. In contrast, the phylogenetic tree constructed with Mega X software [9] based on five concatenated genes (*gyrA, ddl, gdh, rpoB,* and *sodA*) sequences revealed distinct positions of the strains. The phylogenetic analyses deduced from comparisons of concatenated genes position our strain among the streptococci belonging to the Mitis clade [4] and clearly show that it is distinguished from the others (Figure 2). Based on these phylogenetic analyses, we suggest that Marseille-Q2617 is a new member of the genus *Streptococcus*.

### 3.2. Phenotypic Description

**Figure 3:**
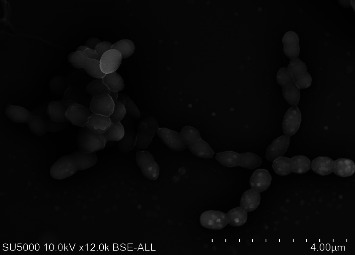
Transmission electron microscopy *Streptococcus thalassemiae* sp. nov. cells are observed on Hitachi SU5000 transmission electron microscope. Scales and magnification are displayed in the figure.

Strain Marseille-Q2617 is a facultative anaerobe growing under aerobic as well as anaerobic conditions at 28 to 42°C but did not grow at 56°C. The optimal growth of this strain is observed at 37°C after 24 hours of incubation in aerobic conditions. The bacterial cells are Gram-positive, coccus shaped, nonmotile, and nonspore forming. The morphology of bacterial cells was revealed with scanning electron microscopy. It displays chain-like structures typical of streptococci (Figure 3). Growth was obtained at all pH levels tested (5.0; 5.5; 6.0; 6.5 and 7.0; and 7.5 and 8). For the salinity test, no growth was recorded.

The results of the API galleries revealed the biochemical characteristics of the strain Marseille-Q2617. Thus, for API ZYM, the positive reactions obtained were for esterase (C4), naphthol-AS-BI-phosphohydrolase and leucine arylamidase, and esterase lipase (C8). An API 50 CH strip showed that strain Marseille-Q2617 was positive for N-acetyl-glucosamine, esculin ferric citrate, D-galactose, D-sucrose, D-tagatose, and potassium 5-ketogluconate. The use of API STREP strips yielded positive reactions following these tests: sodium pyruvate, hippuric acid, esculin ferric citrate, pyroglutamic *ß*-naphthylamide acid, L-leucine-*ß*-naphthylamide, L-arginine, and D-lactose (bovine origin) and starch. The main phenotypic characteristics of strain Marseille-Q2617 were compared with closely related species (Table 1). ETEST® (bioMerieux, Marcy l'Etoile, France) used to determine the minimum inhibitory concentration (MIC) of antibiotics, reveal that strain Marseille-Q2617 is susceptible to penicillin G, amoxicillin, daptomycin, ciprofloxacin, ceftriaxone, clindamycin, doxycycline, fosfomycin, imipenem, linezolid, oxacillin, rifampicin, and teicoplanin but resistant to ceftazidime and trimethoprim‐sulfamethoxazole. Hexadecanoic acid (49.5%) was the major fatty acid detected for Marseille-Q2617 (Table 2).

### 3.3. Genome Features

**Figure 4:**
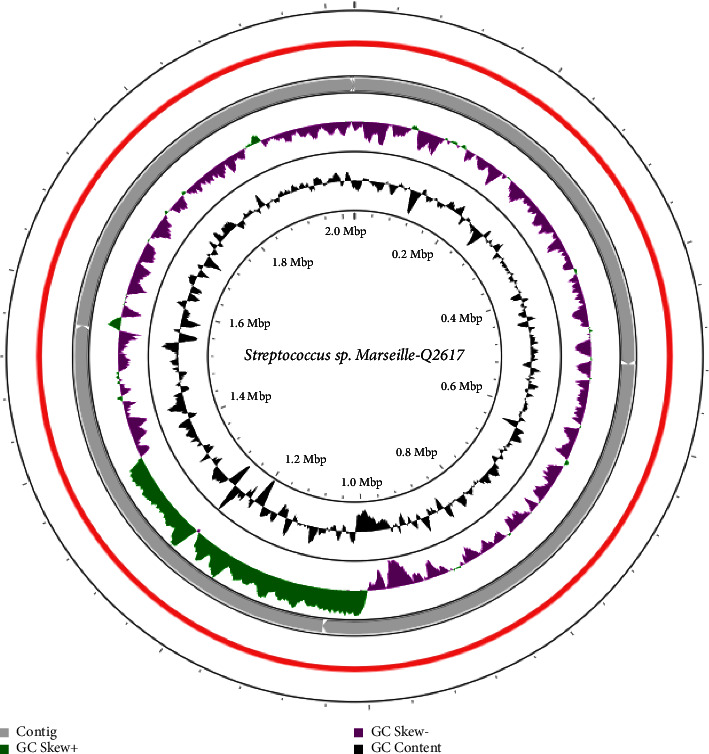
Graphical circular map of the genome of *Streptococcus thalassemiae* sp. nov. Marseille-Q2617 obtained by using the CGView server.

The draft genome size of strain Marseille-Q2617 is 2.02 Mbp long, with a 40.5 mol% G + C content. The genome of strain Marseille-Q2617 consists of 5 contigs and possesses 1 949 predicted genes with 1 779 protein-coding genes and similarly 74 RNA genes (12 rRNAs, 59 tRNAs and 3 other RNA). A total of 96 pseudogenes were detected. The graphical circular map of the draft genomes of Marseille-Q2617 is presented in Figure 4.

The DDH and OrthoANI values obtained are below the threshold values recommended for classifying prokaryotic species [22, 23]. DDH values obtained after genome analysis varied from 22.6% (between *Streptococcus oralis* and *Streptococcus chenjunshii*) to 58.6% (between *Streptococcus pneumoniae* and *Streptococcus pseudopneumoniae*). The strain Marseille-Q2617 shared its highest DDH value (55.4%) with *Streptococcus pseudopneumoniae* (Table 3). Furthermore, in the *Streptococcus* species studied, genomic analysis based on average nucleotide identity (ANI) showed that OrthoANI values ranged from 69.3% (*S. chenjunshii* and *S. pneumoniae*) to 94.7% (*S. pneumoniae* and *S. pseudopneumoniae*). Indeed, the strain Marseille-Q2617^T^ shared the lowest OrthoANI values (69.6%) with the strain *S. chenjunshii*. In addition, the highest OrthoANI values were 94.1% for the strain Marseille-Q2617.

## 4. Conclusion

Based on the phylogenetic, genomic, and phenotypic specificities which give the strain Marseille-Q2617 their unique criteria among the known species belonging to *Streptococcus* genus, we propose that they be considered as novel species, named *Streptococcus thalassemiae* sp. nov.

## 5. Description of *Streptococcus thalassemiae* sp. nov

*Streptococcus thalassemiae *(tha.las.se'mi.ae. N.L. gen. n. thalassemiae, refering to the isolation of the bacterial strain from a patient with thalassemia). It is a Gram-positive cocci bacterium, nonmotile, and nonspore forming. Growth is obtained between 25°C and 42°C, with optimal growth at 37°C. The growth of strain Marseille-Q2617 was observed at different pH values (from 5.0 to 8.0) and salt concentrations of up to 2.5% NaCl. The cells have a diameter varying between 1 to 4 *µ*m: no catalase and no oxidase activities. The colonies are small, punctiform, and pale grayish, with a mean diameter of 0.5 mm on blood agar. Strain Marseille-Q2617^T^ exhibits positive reactions for penicillin G, amoxicillin, daptomycin, ciprofloxacin, ceftriaxone, clindamycin, doxycycline, fosfomycin, imipenem, linezolid, oxacillin, rifampicin teicoplanin, and oxacillin. C_16:0_ (56.1%), C_18:0_ (12.7%), and C_18:1n9_ (11.5%) are the major fatty acids detected in the cell wall of *Streptococcus thalassemiae* sp. nov.

The draft genome of strain Marseille-Q2617^T^ was 2.02 Mbp with 40.5 mol% of G + C content. Indeed, the 16S rRNA and genome sequences of strain Marseille-Q2617^T^ are deposited in the GenBank database under accession numbers LR809138 and CAHJXN010000000, respectively.

The type strain of *Streptococcus thalassemiae* sp. nov., is Marseille-Q2617^T^ (=CSUR Q2617 = CECT 30109) and was isolated from a man with major thalassemia.

## Figures and Tables

**Table 1:** Main differential phenotypic criteria.

Characteristics	**1**	**2**	**3**	**4**	**5**	**6**
*β*-Galactosidase	−	+	−	+	+	+
Hippuric acid	+	−	−	−	−	ND
Esculin ferric citrate	+	−	−	*v*	−	ND
D-glucose	−	+	ND	+	ND	ND
D-fructose	−	*v*	ND	+	ND	ND
D-tagatose	+	−	−	−	ND	+
Potassium 5‐ketogluconate	+	−	ND	−	ND	ND
D-trehalose	−	−	−	−	−	+
G + C (mol%)	40.5	39.8	41	41.1	40.2	39.6
Source	Blood	Clinical specimen	Oral cavity	Mouth	Mouth	Sputum

1 represents *Streptococcus thalassemiae *Marseille-Q2617 compared with close *Streptococcus* species; 2 represents *Streptococcus pseudopneumoniae* CCUG 49455^T^; 3 represents *Streptococcus mitis* NS 51^T^; 4 represents *Streptococcus oralis* 35037^T^; 5 represents *Streptococcus gwangjuense* ChDC B345^T^; 6 represents *Streptococcus pneumoniae* 28588^T^. +, positive reaction; −, negative reaction; *v*, variable.

**Table 2:** Cellular fatty acid profiles (%).

Fatty acids	Name	**1**	**2**	**3**	**4**	**5**
C_16:00_	Hexadecanoic acid	56.1	34.2	29.8	35.5	26.7
C_14:00_	Tetradecanoic acid	7.5	11.4	12.3	14.0	24.0
C_18:1n9_	9-octadecenoic acid	11.5	14.8	ND	11.3	ND
C_12:00_	Dodecanoic acid	TR	4.3	6.6	4.9	11.4
C_18:00_	Octadecanoic acid	12.7	12.8	8.5	12.5	5.4
C_18:2n6_	9.12-octadecadienoic acid	4.3	ND	ND	ND	ND

1 represents *Streptococcus thalassemiae *Marseille-Q2617 compared with close *Streptococcus* species; 2 represents *Streptococcus pseudopneumoniae* CCUG 49455^T^; 3 represents *Streptococcus mitis* NS 51; 4 represents *Streptococcus oralis* 35037^T^; 5 represents *Streptococcus pneumoniae* 28588^T^. TR, trace amounts <1%; ND, not detected.

**Table 3:** Double genomic sequence comparison of Marseille-Q2617 with the most closely related *Streptococcus* species.

Species	*Sth* (%)	*Sau* (%)	*Sch* (%)	*Sgw* (%)	*Smi* (%)	*Sor* (%)	*Spn* (%)	*Sps* (%)
*Sth*		**25.1**	**27.1**	**49.2**	**50.2**	**31.5**	**51.6**	**55.4**
*Sau*	75.3		**27.0**	**25.2**	**25.3**	**25.1**	**25.3**	**25.6**
*Sch*	69.6	69.5		**27.1**	**29.0**	**22.6**	**26.4**	**24.8**
*Sgw*	92.3	75.5	69.5		**54.1**	**31.7**	**45.8**	**47.6**
*Smi*	92.9	75.5	69.8	93.8		**31.6**	**46.3**	**48.3**
*Sor*	85.8	75.4	69.5	86.3	86.2		**31.3**	**31.5**
*Spn*	93.2	75.1	69.3	91.6	91.9	85.8		**58.6**
*Sps*	94.1	75.5	69.4	92.7	92.4	86.1	94.7	

Upper right, DDH values computed using GGDC formula 2 (https://www.ggdc.dsmz.de/ggdc.php). Bottom left, OrthoANI values calculated with the OAT software [18]. Sth, *Streptococcus thalassemiae* Marseille-Q2617; Sau, *Streptococcus australis* NCTC 3168^T^; Sch, *Streptococcus chenjunshii* Z15^T^; Sgw, *Streptococcus gwangjuense* ChDC B345^T^; Smi, *Streptococcus mitis* NCTC 12261^T^; Sor, *Streptococcus oralis* ATCC 35037^T^; Spn, *Streptococcus pneumoniae* CCUG 28588^T^; Sps, *Streptococcus pseudopneumoniae* ATCC BAA-960^T^; ANI, Average Nucleotide Identity. The DDH values are displayed in bold in the upper right corner.

## Data Availability

The 16S rRNA and genome sequences of strain Marseille-Q2617T are deposited in the GenBank database under accession numbers LR809138 and CAHJXN010000000, respectively

## References

[1] Krzyściak W., Pluskwa K. K., Jurczak A., Kościelniak D. (2013). The pathogenicity of the Streptococcus genu. *European Journal of Clinical Microbiology and Infectious Diseases*.

[2] Ricaboni D., Mailhe M., Lagier J.-C. (2017). Noncontiguous finished genome sequence and description of *Streptococcus timonensis* sp. nov. isolated from the human stomach. *New Microbes and New Infections*.

[3] Kawamura Y., Hou X. G., Sultana F., Miura H., Ezaki T. (1995). Determination of 16S rRNA sequences of *Streptococcus mitis* and *Streptococcus gordonii* and phylogenetic relationships among members of the genus *Streptococcus*. *International Journal of Systematic Bacteriology*.

[4] Patel S., Gupta R. S. (2018). Robust demarcation of fourteen different species groups within the genus *Streptococcus* based on genome-based phylogenies and molecular signature. *Infection, Genetics and Evolution*.

[5] Ramasamy D., Mishra A. K., Lagier J.-C. (2014). A polyphasic strategy incorporating genomic data for the taxonomic description of novel bacterial species. *International Journal of Systematic and Evolutionary Microbiology*.

[6] Lagier J.-C., Khelaifia S., Alou M. T. (2016). Culture of previously uncultured members of the human gut microbiota by culturomics. *Nat Microbiol*.

[7] Weisburg W. G., Barns S. M., Pelletier D. A., Lane D. J. (1991). 16S ribosomal DNA amplification for phylogenetic study. *Journal of Bacteriology*.

[8] Morel A.-S., Dubourg G., Prudent E. (2015). Complementarity between targeted real-time specific PCR and conventional broad-range 16S rDNA PCR in the syndrome-driven diagnosis of infectious diseases. *European Journal of Clinical Microbiology and Infectious Diseases*.

[9] Kumar S., Stecher G., Li M., Knyaz C., Tamura K. (2018). Mega X: molecular evolutionary genetics analysis across computing platforms. *Molecular Biology and Evolution*.

[10] Diop A., Diop K., Tomei E. (2019). *Collinsella vaginalis* sp. nov. strain Marseille-P2666T, a new member of the Collinsella genus isolated from the genital tract of a patient suffering from bacterial vaginosis. *International Journal of Systematic and Evolutionary Microbiology*.

[11] Elsawi Z., Togo A. H., Beye M. (2017). *Hugonella massiliensis* gen. nov., sp. nov., genome sequence, and description of a new strictly anaerobic bacterium isolated from the human gu. *Microbiologica*.

[12] Sasser M. (2006). *Bacterial Identification by Gas Chromatographic Analysis of Fatty Acids Methyl Esters (GC-FAME)*.

[13] Dione N., Sankar S. A., Lagier J.-C. (2016). Genome sequence and description of *Anaerosalibacter massiliensis* sp. nov. *New Microbes and New Infections*.

[14] Lo C. I., Traore S. I., Diop A. (2022). *Arabiibacter massiliensis* gen. Nov. sp. nov., new anaerobic bacterium isolated from the human gut. *Current Microbiology*.

[15] Bankevich A., Nurk S., Antipov D. (2012). SPAdes: a new genome assembly algorithm and its applications to single-cell sequencing. *Journal of Computational Biology*.

[16] Grin I., Linke D. (2011). GCView: the genomic context viewer for protein homology searches. *Nucleic Acids Research*.

[17] Meier-Kolthoff J. P., Carbasse J. S., Peinado-Olarte R. L., Göker M. (2021). TYGS and LPSN: a database tandem for fast and reliable genome-based classification and nomenclature of prokaryotes. *Nucleic Acids Research*.

[18] Lee I., Ouk Kim Y., Park S.-C., Chun J. (2016). OrthoANI: an improved algorithm and software for calculating average nucleotide identity. *International Journal of Systematic and Evolutionary Microbiology*.

[19] Kosecka-Strojek M., Wolska M., Żabicka D., Sadowy E., Międzobrodzki J. (2020). Identification of clinically relevant *Streptococcus* and *Enterococcus* species based on biochemical methods and 16S rRNA, sodA, tuf, rpoB, and recA gene sequencin. *Pathogens*.

[20] Sadowy E., Hryniewicz W. (2020). Identification of *Streptococcus pneumoniae* and other Mitis streptococci: importance of molecular method. *European Journal of Clinical Microbiology & Infectious Diseases*.

[21] Kim M., Oh H.-S., Park S.-C., Chun J. (2019). Towards a taxonomic coherence between average nucleotide identity and 16S rRNA gene sequence similarity for species demarcation of prokaryotes. *International journal of systematic and evolutionary microbiology*.

[22] Meier-Kolthoff J. P., Göker M., Spröer C., Klenk H.-P. (2013). When should a DDH experiment be mandatory in microbial taxonomy?. *Archives of Microbiology*.

[23] Meier-Kolthoff J. P., Klenk H.-P., Göker M. (2014). Taxonomic use of DNA G+C content and DNA–DNA hybridization in the genomic age. *International Journal of Systematic and Evolutionary Microbiology*.

